# Surface Defect Detection of Cabbage Based on Curvature Features of 3D Point Cloud

**DOI:** 10.3389/fpls.2022.942040

**Published:** 2022-07-14

**Authors:** Jin Gu, Yawei Zhang, Yanxin Yin, Ruixue Wang, Junwen Deng, Bin Zhang

**Affiliations:** ^1^College of Engineering, China Agricultural University, Beijing, China; ^2^Research Center of Intelligent Equipment, Beijing Academy of Agriculture and Forestry Sciences, Beijing, China; ^3^National Research Center of Intelligent Equipment for Agriculture, Beijing, China; ^4^Chinese Academy of Agricultural Mechanization Sciences Group Co., Ltd., Beijing, China

**Keywords:** defect detection, cabbage, curvature features, 3D point cloud, depth camera

## Abstract

The dents and cracks of cabbage caused by mechanical damage during transportation have a direct impact on both commercial value and storage time. In this study, a method for surface defect detection of cabbage is proposed based on the curvature feature of the 3D point cloud. First, the red-green-blue (RGB) images and depth images are collected using a RealSense-D455 depth camera for 3D point cloud reconstruction. Then, the region of interest (ROI) is extracted by statistical filtering and Euclidean clustering segmentation algorithm, and the 3D point cloud of cabbage is segmented from background noise. Then, the curvature features of the 3D point cloud are calculated using the estimated normal vector based on the least square plane fitting method. Finally, the curvature threshold is determined according to the curvature characteristic parameters, and the surface defect type and area can be detected. The flat-headed cabbage and round-headed cabbage are selected to test the surface damage of dents and cracks. The test results show that the average detection accuracy of this proposed method is 96.25%, in which, the average detection accuracy of dents is 93.3% and the average detection accuracy of cracks is 96.67%, suggesting high detection accuracy and good adaptability for various cabbages. This study provides important technical support for automatic and non-destructive detection of cabbage surface defects.

## Introduction

As one of the economically important vegetable products, cabbage occupies a crucial position in agricultural products. The dents and cracks of cabbage caused by extrusion and collection during transportation have a direct impact both on the commercial value and storage time (Li and Thomas, 2014). The vegetable non-destructive system is a recent trend for quality evaluation, post-harvest classification, and grading (Fathizadeh et al., 2020; Zhang et al., 2021).

The non-destructive testing means mainly include near-infrared spectroscopy and machine vision. The near-infrared spectroscopy-based non-destructive testing is an advanced method, and hyperspectral reflectance imaging can be used to detect the quality of fresh-cut lettuce (Mo et al., 2017). However, it is limited in engineering applications due to the high cost of equipment and slow data processing process (Lu and Lu, 2017; Chen et al., 2021). Machine vision-based non-destructive testing is fast and of low cost, and it identifies the dents and cracks of vegetables according to the color features, texture features, and geometric features. It has achieved good results in tomato defect detection (da Costa et al., 2020), apple defect detection (Zhang et al., 2017), and litchi surface micro-damage detection (Wang et al., 2016). The machine vision algorithm combined with the deep learning model has predominant robustness in carrot defect detection (Xie et al., 2021). Choosing the appropriate learning algorithm for a specific problem is crucial for vegetable defect detection based on the deep learning algorithm. This particularity makes the deep learning technology can only build a standardized detection system for specific targets (Nturambirwe and Opara, 2020). In recent years, the RGB-D depth cameras represented by Intel RealSense series have developed rapidly. It integrates the RGB images and depth images to provide richer information. On this basis, a 3D point cloud based on destructive testing technology provides a fast, convenient, and applicable solution for target detection and surface 3D reconstruction (Jiang et al., 2018; Wu et al., 2019; Das Choudhury et al., 2020). It has been successfully used to detail road surface defects, composite wrinkle defects, and seamless steel pipe wear defects (Zhang et al., 2018; Hu and Furukawa, 2020; Zong et al., 2021). The 3D point cloud technology has an excellent performance in object detection (Ying et al., 2013). Therefore, it is meaningful to build a cabbage surface defect detection system based on 3D point cloud for presale testing applications.

Curvature is an important basis for feature recognition. The variation of edge curvature of dents and cracks usually fluctuates obviously. In this study, we took the RealSense-D455 depth camera as the sensor to rebuild the 3D point cloud of the image, and the target 3D point cloud of cabbage is segmented from the background noise through preprocessing and region of interest (ROI) extraction. The normal vector is estimated based on the least-squares plane fitting method, and the curvature threshold is defined in agreement with the curvature character parameters. The surface defect detection is realized according to the curvature difference between the normal area and the defective area on the cabbage surface.

## Materials and Methods

### 3D Point Cloud Reconstruction System

**FIGURE 1:**
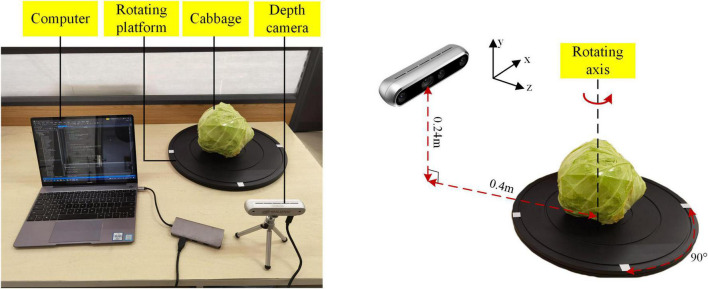
3D point cloud reconstruction system.

The 3D point cloud reconstruction system was built using a RealSense-D455 depth camera, a rotating platform, and a computer, as shown in Figure 1. The RealSense-D455 depth camera was used as a sensor to get a 3D point cloud of the cabbage surface, and its parameters are shown in Table 1. The rotating platform rotated at 45° intervals to record the images from different angles; therefore, for each cabbage, 8 frames of point clouds could be obtained. The computer was used for collecting and analyzing the RGB images and depth images from the camera and reconstructing the 3D point clouds. As the most common cabbage, the flat-headed cabbage and round-headed cabbage were selected to conduct experiments.

**TABLE 1:** Parameters of the D455 depth camera.

Parameters	Values
RGB frame resolution/(pixels)	1280 × 800
Depth output resolution/(pixels)	1280 × 720
RGB frame rate/(frame/s)	30
Depth field of view/(°)	87 × 58
Ideal range/(m)	0.6 ∼ 6
Depth Accuracy	<2% at 4 m

**FIGURE 2:**
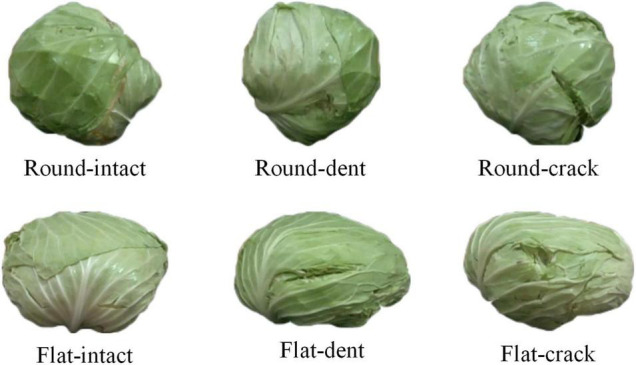
Image of cabbage samples.

To ensure the consistency of cabbage samples, the diameter of the cabbage was in the range of 150–200 mm, and the mass of the cabbage was in the range of 0.8–1.2 kg. The two varieties of cabbage samples contain three types, namely, intact cabbage, crack cabbage, and dent cabbage, as shown in Figure 2. In experiments, the cabbage was placed in the center of the rotating platform. The distance between the camera and the top of the rotating platform was 0.4 m, and the height between the camera and the center of the rotating platform was 0.24 m. The computer is configured as Intel (R) Core (TM) i5-8265 CPU @ 1.6 GHz, 8 GB RAM, NVIDIA GeForce MX150 graphics card. All algorithms of surface defect detection method were written in C++, using RealSense SDK 2.0 provided by Intel Corporation and open source library PCL 1.8.1.

### Point Cloud Denoising

**FIGURE 3:**
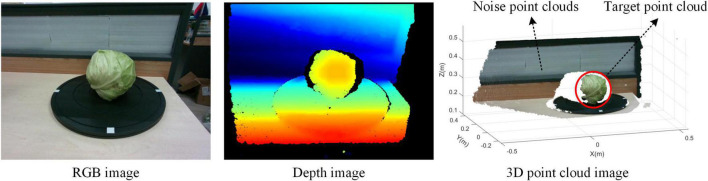
Image fusion.

The camera fuses the collected RGB images with the depth images to obtain the point cloud data in the view (Condotta et al., 2020). Figure 3 shows the RGB image of the cabbage in one view, the corresponding depth image, and the 3D point cloud image after aligning and fusing the RGB image with the depth image.

**FIGURE 4:**
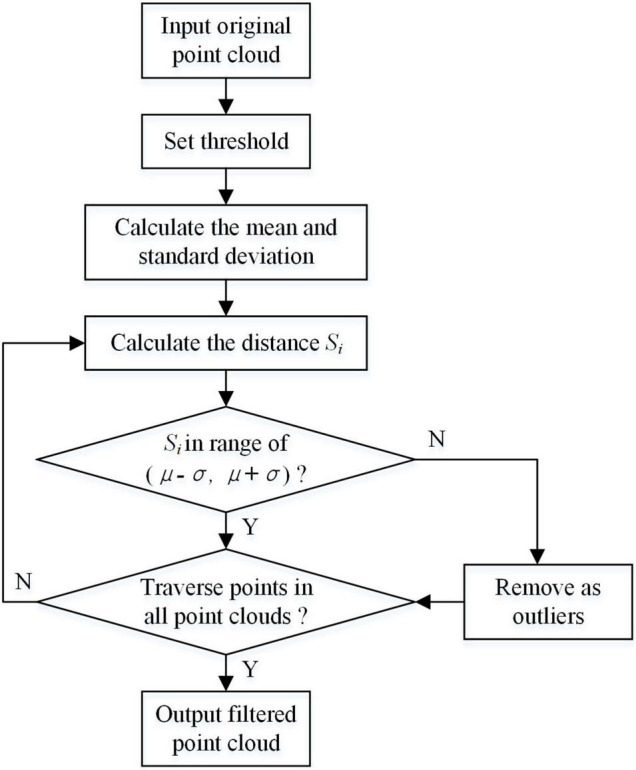
Flowchart of statistical filtering.

The original point cloud contains a large number of background noises and redundant outliers, which affects the processing effect of subsequent clustering segmentation. In this study, statistical filters were used to remove outliers with sparse edge distribution of point cloud. The principle of statistical filtering is to calculate the average value of the distances from each point to the points in the neighbor according to the sparse degree of points in space. Then, the points whose average distance is outside the standard range are removed. Figure 4 shows the point cloud statistical filtering process.

First, the average value of distances from each point to *i* points in the neighbor was calculated. The average values constituted a Gaussian distribution, and its shape was determined by mean value μ and standard deviation σ. The coordinate of one point was removed *Pn*(*Xn*,*Yn*,*Zn*). The distance from the point *Pn*(*Xn*,*Yn*,*Zn*) to the other point *Pm*(*Xm*,*Ym*,*Zm*) could be calculated as follows:


(1) $$S_{i}=\sqrt{{\left(X_{n}-X_{m}\right)}^{2}+{\left(Y_{n}-Y_{m}\right)}^{2}+{\left(Z_{n}-Z_{m}\right)}^{2}}$$

The average value of the distances between all the points could be calculated as follows:


(2) $$\mu =\frac{1}{n}\,\sum_{i=1}^{n}S_{i}$$

The standard deviation of distance could be calculated as follows:


(3) $$\sigma =\sqrt{\frac{1}{n}\,\sum_{i=1}^{n}{\left(S_{i}-\mu \right)}^{2}}$$

**FIGURE 5:**
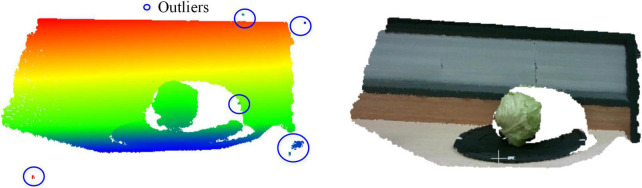
Statistical filtering result of the point cloud.

When the average value of distances was beyond the range (μ−σ,μ + σ), this point is regarded as an outlier. In this algorithm, *i* was set to the threshold. When *i* = 50, the point cloud after statistical filtering is shown in Figure 5. The points in blue circles are outliers. The outliers were effectively eliminated from the origin point cloud after statistical filtering.

### Extract the Region of Interest

To extract the point cloud of target cabbage from the point cloud containing background information, it is necessary to segment the point cloud data to extract the ROI. The principle of point cloud clustering segmentation is to classify the point cloud according to geometric and texture features. The point clouds with similar features are clustered into one class. Common point cloud segmentation methods are the Random Sampling Consistency (RANSAC) algorithm, region growing algorithm, and Euclidean clustering segmentation algorithm (Xu et al., 2015; Wu et al., 2020; Luo et al., 2021). Considering processing time and segmentation effect, the Euclidean clustering segmentation algorithm was used to extract the ROI of the point cloud.

The Euclidean clustering segmentation algorithm is a distance measure clustering algorithm based on k-dimensional (KD)-tree nearest neighbor search, which takes Euclidean distance as the judgment criterion (Li et al., 2021).

Step 1: For a point *p*, the *n* points of the nearest neighbor by KD-tree were searched, and the distances between the points to the point *p* were calculated, respectively.


(4) $$d_{i}\,\left(p,q_{i}\right)=\sqrt{\sum_{i=1}^{n}{\left(p-q_{i}\right)}^{2}}$$

Step 2: The threshold was set to *r*. If the distance *di* was less than the set threshold *r*, the point *qi* was clustered in the database *Q*. Until the number of points in *Q* no longer increased, indicating that the clustering of points in this category is completed.

Step 3: Continue to select another point in the space and repeat the above operation, until the number of point cloud category sets was no longer increasing.

**FIGURE 6:**
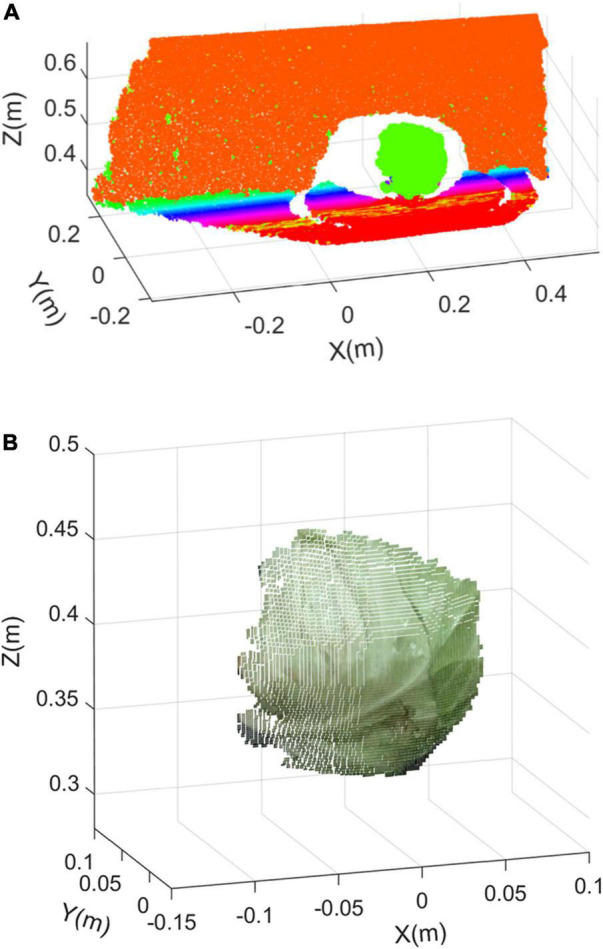
Clustering segmentation extraction result: **(A)** result of the European clustering segmentation algorithm and **(B)** the target cabbage point cloud.

Figure 6A shows the point cloud clustering results using the European clustering segmentation algorithm. Figure 6B shows the target cabbage point cloud. It can be seen that the target cabbage is effectively clustered from the background noises, and the processing time of this algorithm was 3.648 s.

### Point Cloud Subsampling

The point cloud after clustering segmentation extraction contained a mass of data. To speed up the subsequent processing, it was necessary to minimize the amount of data without losing point cloud features. In this study, a voxel filter was used to subsample the point cloud of cabbage.

**FIGURE 7:**
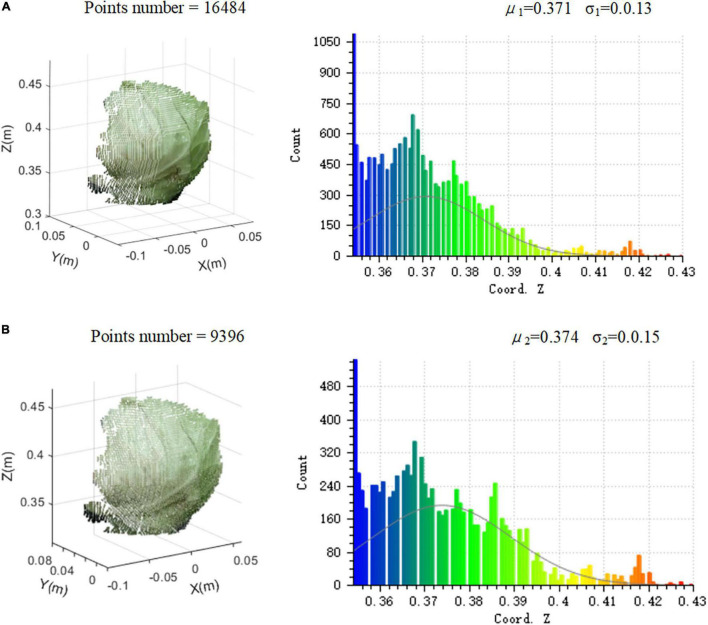
The point distribution histogram before and after subsampling: **(A)** the point cloud before subsampling and **(B)** the point cloud after subsampling.

The principle of the algorithm for the voxel filter was to create tiny three-dimensional cubes in space, namely, voxel grid, and divide the point cloud using the voxel grid (Xiong et al., 2021). In each voxel, the centroid of all the points was used to replace all the points to minimize the amount of point cloud. The size of the three-dimensional voxel grid was set to 1 mm. Figure 7 shows the point distribution histogram of a frame of cabbage before and after subsampling.

Figure 7A shows the point cloud before subsampling, which contains 16,484 points in total. The distribution on the *Z*-axis of all the points is expressed as Gaussian distribution, with the mean value of μ1 = 0.371 and the standard deviation of σ_1_ = 0.013. Figure 7B shows the point cloud after subsampling, and the number of points drops to 9,396. The distribution on the *Z*-axis of all the points is expressed as the Gaussian distribution, with the mean value of μ_2_ = 0.374 and the standard deviation of σ2 = 0.015 after subsampling. From Gaussian distributions, it can be seen that the rate of changes in mean value and the standard deviation are less than 2% when the number of point clouds decreases by 42.9%. It shows that the spatial distribution characteristics of point clouds before and after subsampling are very similar. Therefore, it can be concluded that the selection of the voxel filter grid size is relatively accurate.

### Cabbage Surface Defect Detection Method

#### Normal Vector Estimation and Curvature Calculation

Surface curvature describes the change degree of point cloud by the eigenvalue. The curvature at a certain point can be obtained by estimating the normal vector of each point in the point cloud. The normal vector can be calculated using the normal of the tangent line of the surface at that point. According to the least square method (LSM), the quadratic surface can be used to characterize the local region, and the normal vector can be estimated using the local region surface fitting method.

Each point in the point cloud has a neighborhood point cloud, which is approached using a certain surface. The curvature of a certain point can be expressed by the curvature of the local surface fitted by the point and its neighborhood points. By taking the point *Pi* as a central point, *k* points were uniformly selected in the neighborhood of point *Pi*.

The quadratic surface equation can be expressed as follows:


(5) $$z\,\left(x,y\right)=a\,x^{2}+b\,x\,y+c\,y^{2}$$

According to the principle of least squares, the sum of the squares of *zi* can be expressed as follows:


(6) $$Q^{2}=\sum_{i}{\left(ax_{i}^{2}+bx_{i}y_{i}+cy_{i}^{2}=z_{i}\right)}^{2},j\in \left(0,k\right)$$

where, *xi*, *yi*, and *zi* are points in the neighbor of point *Pi*. The derivative of the coefficient is obtained in Eq. 6.


(7) $$\left\{ \begin{array}{c} \frac{\partial Q^{2}}{\partial a}=\sum_{i}2\,x_{i}^{2}\,\left(a\,x_{i}^{2}+b\,x_{i}\,y_{i}+c\,y_{i}^{2}-z_{i}\right)=0\\ \frac{\partial Q^{2}}{\partial b}=\sum_{i}2\,x_{i}\,y_{i}\,\left(a\,x_{i}^{2}+b\,x_{i}\,y_{i}+c\,y_{i}^{2}-z_{i}\right)=0\\ \frac{\partial Q^{2}}{\partial c}=\sum_{i}2\,y_{i}^{2}\,\left(a\,x_{i}^{2}+b\,x_{i}\,y_{i}+c\,y_{i}^{2}-z_{i}\right)=0 \end{array}\right.,i\in \left(0,k\right)$$

From this, the values of the coefficients *a*, *b*, and *c* of the quadratic surface equation can be solved. Equation 6 is expressed as a parametric form.


(8) $$r\,\left(x,y\right)=\left\{ \begin{array}{c} X\,\left(x,y\right)=x\\ Y\,\left(x,y\right)=y\\ Z\,\left(x,y\right)=a\,x^{2}+b\,x\,y+c\,y^{2} \end{array}\right.$$

Then, a curve on the surface can be expressed as follows:


(9) r=r⁢(x⁢(t),y⁢(t))

The arc length differential equation of the curve can be obtained by derivation.


(10) $${\left(d\,s\right)}^{2}=r_{x}^{2}\,{\left(d\,x\right)}^{2}+2\,r_{x}\,r_{y}\,d\,x\,d\,y+r_{y}^{2}\,{\left(d\,y\right)}^{2}$$

The unit normal vector at point *Pi* can be expressed as follows:


(11) $$n_{i}=\frac{r_{x}\times r_{y}}{\left|r_{x}\times r_{y}\right|}$$

Considering the normal vector at the point *Pi* as the normal vector of the local surface in the neighborhood, then the covariance matrix of the points in the neighborhood was as follows:


(12) $$C=\frac{1}{k}\,\sum_{i=1}^{k}\left(P_{i}-P_{0}\right)\,{\left(P_{i}-P_{0}\right)}^{T}$$

(13) $$C\cdot X_{j}=\lambda_{j}\cdot X_{j},j=1,2,3$$

where *P*0 is the centroid of points in the neighborhood, *k* is the number of points in the neighborhood, and λ*j* and *Xj* represent the eigenvalue and eigenvector of *C*, respectively.

The eigenvector corresponding to the smallest eigenvalue of matrix *C* was the normal vector of point *Pi*. The covariance matrix was constructed, and the eigenvalues of the matrix were calculated by eigenvalue decomposition.

If the eigenvalues satisfied the condition λ0≤λ1≤λ2, the curvature of the neighborhood points can be expressed as follows:


(14) $$\kappa_{i}=\frac{\lambda_{0}}{\lambda_{0}+\lambda_{1}+\lambda_{2}}$$

Neighborhood curvature represents the degree of curvature of the neighborhood surface. The curvature values of all points in the point cloud are calculated. The curvature features were used to determine the surface concavity of the cabbage point cloud to improve the accuracy of damage detection.

#### Cabbage Defect Detection Algorithm Based on Curvature

Cabbage surface damage generally manifests itself in the form of dents and cracks. The dent damage is mainly due to extrusion or falling, and the edges are generally round or oval with shallow internal depressions. The crack damage is mainly due to growth cracking or scratching by sharp objects, which leads to multilayered fracture of cabbage. The edges are generally narrow-shaped and have deep internal depressions. Since the curvature of the edges of the damaged area would change significantly compared with the normal area, the damaged area could be extracted according to the surface type represented by different curvatures.

**FIGURE 8:**
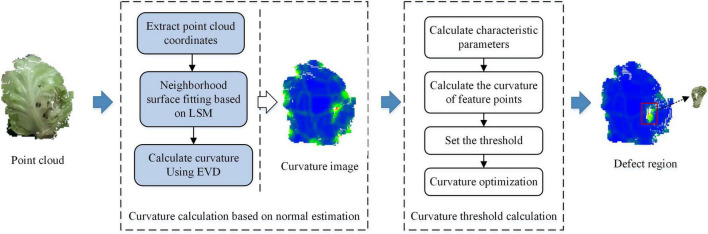
Defective area extraction process.

Taking one frame of cabbage point clouds as an example, Figure 8 shows the extraction process of cabbage surface defective area. It can be seen from the curvature image that the curvature of the edge of the damaged area is significantly higher than that of the normal area of cabbage, so the defective area can be extracted by setting the curvature threshold and segmenting the curvature of the point cloud according to the threshold.

From the curvature calculation results, it can be seen that although the cabbage curvature image shows the surface damaged area, it is mistakenly segmented as a damaged area due to the curvature gradient at the edge of the point cloud. However, the curvature change caused by the protrusion of cabbage leaf stalk and cabbage itself dents will also interfere with the detection results. To solve these problems, the defect extraction results can be segmented twice by optimizing the curvature threshold.

According to the definition proposed by Chen and Bhanu (2007), the point with the largest curvature change on the surface of the point cloud is considered the feature point. Whether the current point*P*_*i*_ is a feature point should be checked. The formula for the curvature characteristic parameter can be expressed as follows:


(15) $$S\,\left(P_{i}\right)=\frac{1}{2}-\frac{1}{\pi }\,\arctan\frac{k_{1}\,\left(P_{i}\right)+k_{2}\,\left(P_{i}\right)}{k_{1}\,\left(P_{i}\right)-k_{2}\,\left(P_{i}\right)}$$

where *k*1 and *k*2 are the maximum principal curvature and the minimum principal curvature, respectively, which can be obtained by calculating the two roots of normal curvature according to the second basic formula of the surface.

The criteria to determine whether the current sampling point is a feature point is as follows: if *S*(*Pi*) > *max*⁡(*S*(*Pi*1),*S*(*Pi*2),…,*S*(*Pik*)), the point *Pi* is a curvature feature point.

The curvature parameter at the feature points was taken as the threshold value, and the final point cloud that satisfies the conditions was extracted as the correct damaged area. It can be seen from the extracted image of the cabbage damaged area that this method effectively eliminates the influence of curvature change caused by the shape of the cabbage surface on the test results and accurately extracts the damaged area of the cabbage.

## Results and Discussion

### Accuracy Analysis of Point Cloud Detection Methods

**FIGURE 9:**
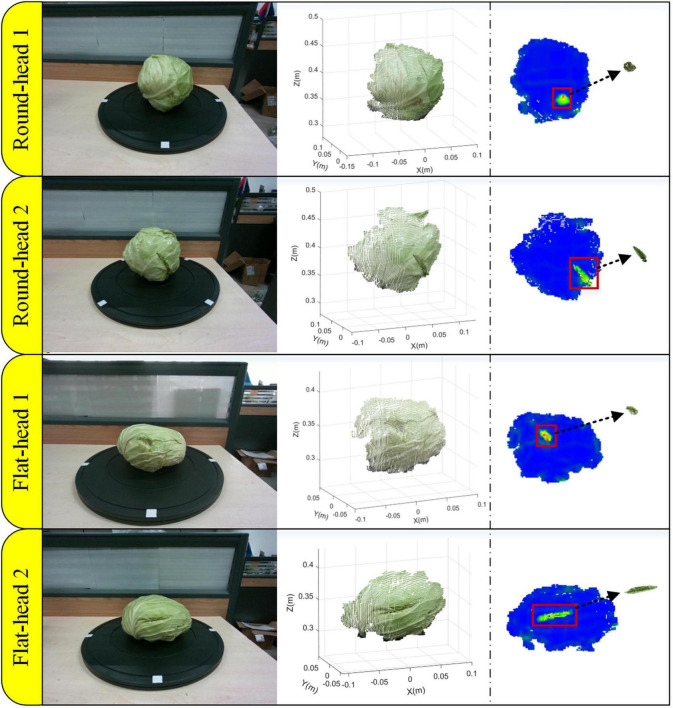
Defective area extraction results.

The detection effect of this algorithm was evaluated by selecting different varieties and different defect types of cabbage samples. Figure 9 shows four visualization results of this method, including round-headed cabbage dent damage, round-headed cabbage crack damage, flat-headed cabbage dent damage, and flat-headed cabbage crack damage. The original cabbage image, the point cloud image including the defects’ part, the curvature threshold calculation result, and the defect point cloud extraction result are, respectively, shown in the figure. Based on the curvature statistics of the point cloud samples, the curvature threshold was determined as 0.08. The point cloud area of cracking damage is larger than that of denting damage, but the curvature mutation of denting damage is more obvious. It shows that the algorithm has good adaptability to two varieties and two damaged types of cabbage.

To evaluate the performance of the proposed algorithm in detecting different types of cabbage damage, 80 cabbage point cloud samples collected were tested for detection. It includes 30 samples of dent damage (15 samples per variety), 30 samples of crack damage (15 samples per variety), and 20 intact samples (10 samples per variety). The correct classification accuracies were calculated and evaluated for dent damaged samples, crack damaged samples, and intact samples of different varieties of cabbage, respectively, and the results are shown in Table 2.

**TABLE 2:** Detection results of point cloud defect detection method.

Samples	Types	Number	Correct detection	Accuracy (%)
Denting cabbages	Round-headed	15	14	93.3
	Flat-headed	15	14	93.3
Cracking cabbages	Round-headed	15	15	100
	Flat-headed	15	14	93.3
Intact cabbages	Round-headed	10	10	100
	Flat-headed	10	10	100
Total	6	80	77	96.25

Table 2 shows the test results of all samples. In terms of dent damage detection, the accuracy of the point cloud detection method for round-headed cabbage and flat-headed cabbage is 93.3%. In terms of crack damage detection, the detection accuracy of the point cloud detection method for round-headed cabbage is 100%, that of flat-headed cabbage is 93.3%, and the average detection accuracy of the two varieties is 96.67%. The detection accuracy of cracked damaged samples is higher than that of dented damaged samples. From the overall test results, the average detection accuracy of the point cloud detection method is 96.25%, and it appears that this method could effectively detect dent-damaged cabbage, crack-damaged cabbage, and intact cabbage samples. The detection accuracy of the intact cabbages is 100%, indicating that the detection method can distinguish the cabbage itself dents from other defects, and the detection error of intact cabbage is low. The detection error in the test mainly occurs in the detection of defective samples, and the detection accuracy of the defect cabbages is 95%. It indicates that this method has good detection performance.

### Misjudgment Analysis of Detection Results

**FIGURE 10:**
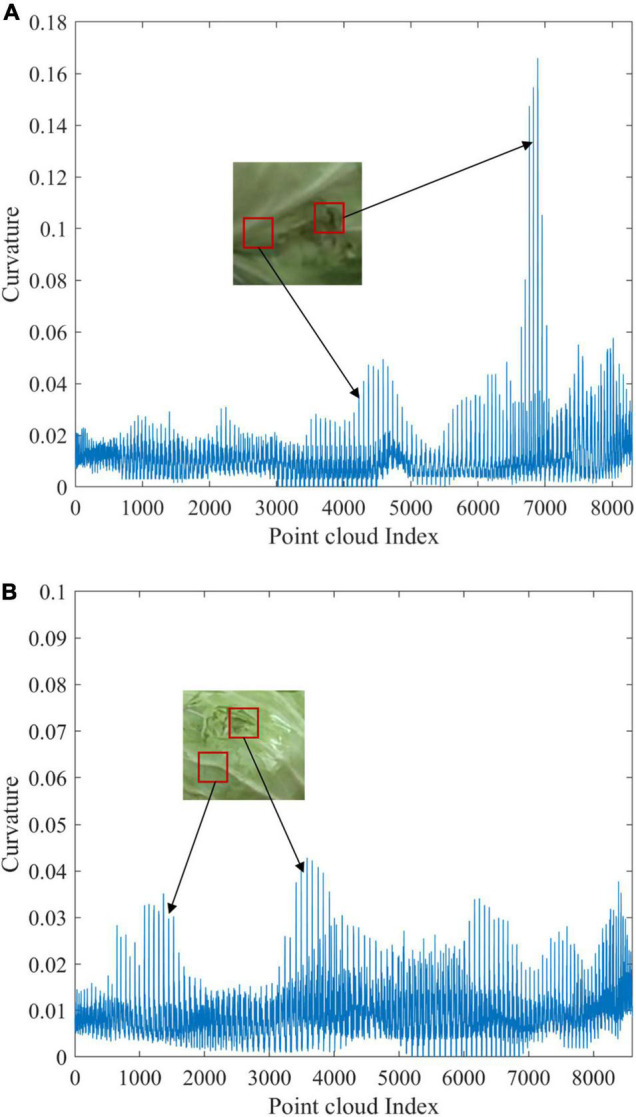
Curvature curves of dent detection of cabbage: **(A)** the curvature curve of a correctly detected dent sample and **(B)** the curvature curve of a dent sample misjudged as non-maging.

To further analyze the reasons for the higher misjudgment rate of dent samples, the curvature calculation results of representative cabbage sample point clouds were extracted and represented as curvature curves according to the point cloud index values. Figure 10 shows the comparison results.

Figure 10A shows the curvature curve of a correctly detected dent sample, and Figure 10B shows the curvature curve of a dent sample misjudged as non-damaging. It can be seen that the curvature of the defective area varies less due to the smaller area and shallow depth of the depressed area of the sample in Figure 10B. The extreme value of curvature is 0.043, which is very close to the curvature size of interference regions such as leaf stems, and it is difficult to be accurately segmented by a single curvature threshold so the correct detection rate is low. In contrast, the area of damage in the depressed sample in Figure 10A is larger, the abrupt change of edge curvature is more obvious, the extreme value of curvature in the defective area reaches 0.167, and the surface curvature distribution of other parts is relatively uniform except for the defective area. The curvature fluctuation mainly appears at the leaf stem position, in which the maximum value of curvature is 0.059, much lower than that in the defective area. Therefore, the defective area can be easily extracted by curvature threshold segmentation.

**FIGURE 11:**
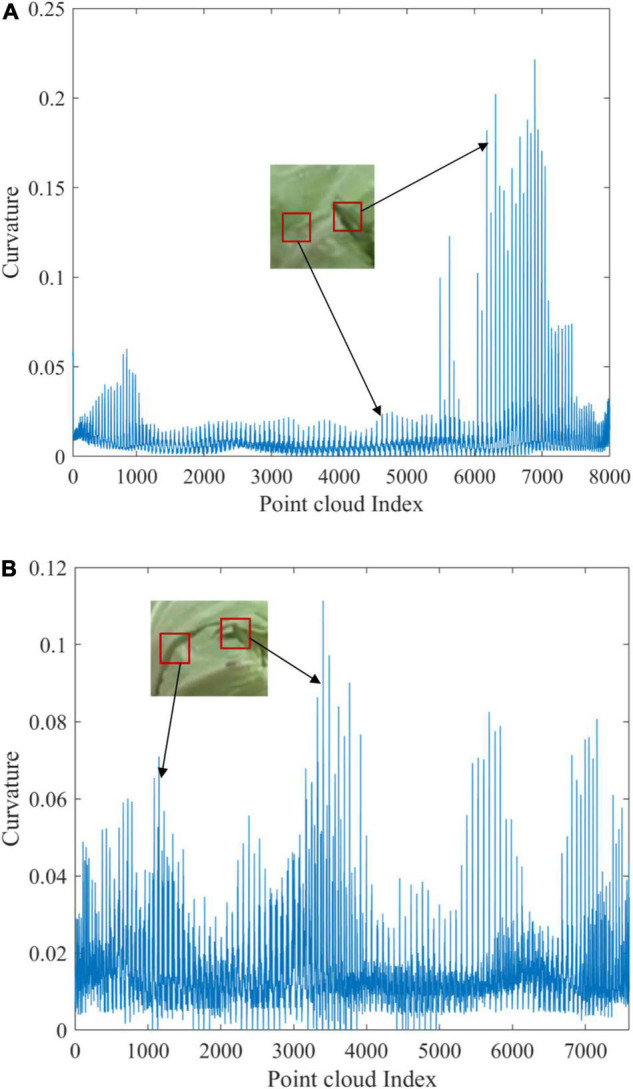
Curvature curves of different variables of cabbage: **(A)** the curvature curve of the cracked sample of round-headed cabbage and **(B)** the curvature curve of the cracked sample of flat-headed cabbage.

It is also found that different varieties of cabbage had differences in detection accuracy in this study. According to the point cloud detection results of all defect types, the average detection accuracy of round-headed cabbage is 97.5%, and that of flat-headed cabbage is 95%. It is inferred that the cabbage type may be one of the reasons affecting the detection accuracy. To further analyze the reasons for the misjudgment, the curvature calculation results of representative cabbage sample point clouds were extracted and represented as curvature curves according to the point cloud index values. Figure 11 shows the comparison results.

Figure 11A shows the curvature curve of the cracked sample of round-headed cabbage, and Figure 11B shows the curvature curve of the cracked sample of flat-headed cabbage. By analyzing the point cloud curvature curve of the two varieties of cabbage, it was concluded that the difference in detection accuracy was caused by the difference in the surface shape of the cabbage. From Figure 11A, it can be seen that the curvature fluctuation is smaller in the undamaged area due to the tighter wrapping of round-headed cabbage, while the cracked area usually has a more pronounced edge curvature mutation. The curvature extreme value of the selected sample in the cracked area is 0.227, which is much higher than that in the undamaged area. As for Figure 11B, the curvature of flat-headed cabbage fluctuates greatly as a whole, mainly because there are many leaf edge bulges forming curvature mutation, which makes the curvature value of these areas close to the defective area, making the detection algorithm easy to misjudge and causing the recognition rate to decrease.

## Conclusion

To provide an effective means of detecting mechanical damages such as dents and cracks on the surface of cabbage, this study proposes a method for surface defect detection of cabbage that is proposed based on the curvature feature of the 3D point cloud. The experimental results show that this method has high accuracy and good robustness in detecting surface depression damage and cracking damage of flat-headed cabbage and round-headed cabbage. The conclusion of this study were as follows:

1. The cabbage point cloud collection platform was built based on the D455 depth camera. The ROI of cabbage samples was extracted by statistical filtering and the European clustering segmentation method, and the invalid information of the point cloud was removed. The data amount of point cloud was reduced by voxel down sampling. Based on the least square plane method, the normal vector was estimated, and the curvature of the point cloud was calculated. According to the curvature characteristic parameters, the curvature threshold was determined, and the interference factors on the surface of the cabbage were eliminated to accurately obtain the defective area.
2. In total, 80 point cloud samples of cabbage (including flat-headed cabbage and round-headed cabbage) were tested in the laboratory. It includes 30 samples of dent damage, 30 samples of crack damage, and 20 intact samples. The experimental results show that the average detection accuracy of the point cloud detection method for 80 samples was 96.25%. The average detection accuracy was 93.3% for dent cabbage samples and 96.67% for crack cabbage samples. In terms of the detection effect of two varieties of cabbage, the average detection accuracy of this method for round-headed cabbage was 97.5%, and the average detection accuracy of flat-headed cabbage was 95%. The results showed that the method had an excellent detection effect on two varieties and two defect types of cabbage.
3. In this study, a consumer-grade D455 depth camera was used to detect the defective area of cabbage, which provided an inexpensive automatic solution for the cabbage quality screening. In some cases, the detection accuracy decreased when the leaf edge bumps and dents were shallow. It is difficult to completely solve the problem by a single detection means. In the future, the detection accuracy and robustness can be improved by enhancing the hardware acquisition accuracy and developing the fusion of multiple detection means and multi-threshold discrimination.

## Data Availability Statement

The original contributions presented in this study are included in the article/supplementary material, further inquiries can be directed to the corresponding author.

## Author Contributions

JG, YZ, RW, and JD built the system, conducted experiments, and wrote the manuscript. BZ and YY designed the detection method. All authors discussed the detection method, designed the experiments, contributed to the article, and approved the submitted version.

## Conflict of Interest

RW was employed by Chinese Academy of Agricultural Mechanization Sciences Group Co., Ltd. The remaining authors declare that the research was conducted in the absence of any commercial or financial relationships that could be construed as a potential conflict of interest.

## Publisher’s Note

All claims expressed in this article are solely those of the authors and do not necessarily represent those of their affiliated organizations, or those of the publisher, the editors and the reviewers. Any product that may be evaluated in this article, or claim that may be made by its manufacturer, is not guaranteed or endorsed by the publisher.
